# Total Abdominal Hysterectomy

**Published:** 1897-06

**Authors:** S. S. Prosser

**Affiliations:** Savannah, Ga.


### Southern Medical Record.

A MONTHLY JOURNAL OF MEDICINE AND SURGERY.

Vol. XXVII. ATLANTA, GA., JUNE, 1897. No. 5 6

### Original Articles.

### TOTAL ABDOMINAL HYSTERECTOMY.

By S. S. PROSSER, M. D., Savannah, Ga.

In presenting the details of this operation the writer is impelled by the thought that the subject may be of some interest in these days when our surgeons are so deeply interested in abdominal surgery.

Hattie Warren, aged thirty-eight, presented herself at my office on March 27, last, and stated that she had been aware for the past ten years of a gradual development of an abdominal growth and which during the last eight months had resulted in almost total disability. Her abdomen was distended to a greater extent than a full term pregnancy.

Bi-manual examination showed the uterus to be greatly enlarged and that it had become part of the tumor.

Interuterine examination proved the uterine cavity to be about six inches and on palpation a pedunculated tumor was located and found to be movable from side to side of the abdomen.

The patient was informed that it would be necessary to perform an abdominal hysterectomy and the seriousness of such an operation was fully explained. She readily consented and accordingly on Sunday, April 11, aided only by a student to whom was entrusted the anesthetizing and with two female nurses as attendants, I proceeded with the operation.

Placing the patient in the Trendelenburg posture, an incision was made through the abdomen beginning immediately below the umbilicus and extending to the pubes. It was then found that the tumor had adhered to the greater omentum. Upon the interuterine tumor which weighed ten pounds nine ounces including the uterine appendages, was attached a pedunculated tumor weighing five pounds, four ounces.

With a pair of tumor forceps the whole mass was lifted towards the opening, sponges being packed against the intestines as a protection and to prevent any protrusion.

The pedunculated tumor was then seized and drawn through the abdominal opening and its greater omentum attachments ligated and severed. The interstitial tumor was then grasped by forceps, traction used, but it was found impossible to draw it through the incision. A pair of vaginal hysterectomy clamps was then placed on the broad ligament on the right side and three double ligatures of heavy braided silk were put through the broad ligament including the ovarian artery. The broad ligament was then severed leaving the ovary and tube attached to the tumor.

Owing to the extensive growth of the mass it was impossible to ligate on the left side, but by forcing the tumor over as much as possible, clamps were placed on the broad ligament close to the tumor and the broad ligament divided between the clamps and the tumor, thus leaving behind the left ovary and tube. The tumor was then raised from its bed as high as possible and double ligatures of silk run through the broad ligament and tube. Having now secured the ovarian artery, the division of the broad ligament was continued down to the utero-vesical pouch and an incision made across the uterus in the front and to a corresponding point on the opposite side.

The peritoneal flap was then dissected to the cervico-vaginal junction. A similar incision was made around the posterior aspect of the uterus and the flap dissected back releasing the utero-rectal attachments. Much difficulty was experienced by the operator while carrying out this procedure in ligating the uterine arteries and protecting the uterus. The uterine arteries having been ligated between the cervix and the ureters, a semicircular incision was made around the cervix and the tumor amputated. The pedunculated and interstitial tumors with the right tube and ovary attached were then removed en masse.

The ligature that had been applied to the broad ligament on the left side was then brought forward, traction being used, and the broad ligament ligated externally to the tube and ovary, three ligatures being applied to cut off anastomosis, and the tube and ovary removed. The cervical flaps which had been formed were now brought together by a continuous silk suture closing the cervix and whipping together by the same suture the cut edges of the peritoneum, leaving the cervix, the uterine and ovarian ligatures all extraperitoneal.

The sponges were then removed and the peritoneal cavity thoroughly cleansed with a warm bi-chloride solution. The abdominal wound was closed with a row of buried sutures, over which was run a continuous suture, no drainage being used. I then packed the vagina with sterilized gauze and applied a comfortable amount of the same to the vulva. Over the abdominal wound was sprinkled a mixture of iodoform and acetanilide, then a layer of bichloride gauze on top of which was laid a pad of absorbent cotton, the whole being held in place by strips of adhesive plaster and a many tail bandage.

When taken from the table and placed on the bed, the patient was in very fair condition, having been under the anesthetic two hours and five minutes. The recovery was almost without event of any significance. The next morning her temperature was one degree above normal, and that afternoon a heavy sweat with some signs of collapse, which occurred again the following morning; after which the thermometer registered normal.

The only difficulty that occurred throughout the period of recovery was in securing a movement of the bowels and a stitch-track abscess that made its appearance on the sixth day. For the first three days the bowels resisted the saline purgatives that were administered, but a healthy evacuation was obtained on the morning of the fourth day and they have since been regular. The stitch-track abscess was emptied daily for three days, and no further trouble has been encountered. Fourteen days have now elapsed and the patient is in excellent condition, enjoying her usual diet, and from present indications will be allowed to leave her room on the twentieth day.

